# Modeling and optimization study on degradation of organic contaminants using nZVI activated persulfate based on response surface methodology and artificial neural network: a case study of benzene as the model pollutant

**DOI:** 10.3389/fchem.2023.1270730

**Published:** 2023-10-19

**Authors:** Moye Luo, Xiaodong Zhang, Tao Long, Sheng Chen, Manjun Zhan, Xin Zhu, Ran Yu

**Affiliations:** ^1^ Department of Environmental Science and Engineering, School of Energy and Environment, Southeast University, Nanjing, China; ^2^ State Environmental Protection Key Laboratory of Soil Environmental Management and Pollution Control, Nanjing Institute of Environmental Sciences, Ministry of Ecology and Environment, Nanjing, China; ^3^ Geo-engineering Investigation Institute of Jiangsu Province, Nanjing, China; ^4^ Nanjing Research Institute of Environmental Protection, Nanjing Environmental Protection Bureau, Nanjing, China

**Keywords:** benzene, activated persulfate, response surface methodology, artificial neural network, kinetics

## Abstract

Due to the complicated transport and reactive behavior of organic contamination in groundwater, the development of mathematical models to aid field remediation planning and implementation attracts increasing attentions. In this study, the approach coupling response surface methodology (RSM), artificial neural networks (ANN), and kinetic models was implemented to model the degradation effects of nano-zero-valent iron (nZVI) activated persulfate (PS) systems on benzene, a common organic pollutant in groundwater. The proposed model was applied to optimize the process parameters in order to help predict the effects of multiple factors on benzene degradation rate. Meanwhile, the chemical oxidation kinetics was developed based on batch experiments under the optimized reaction conditions to predict the temporal degradation of benzene. The results indicated that benzene (0.25 mmol) would be theoretically completely oxidized in 1.45 mM PS with the PS/nZVI molar ratio of 4:1 at pH 3.9°C and 21.9 C. The RSM model predicted well the effects of the four factors on benzene degradation rate (R^2^ = 0.948), and the ANN with a hidden layer structure of [8-8] performed better compared to the RSM (R^2^ = 0.980). In addition, the involved benzene degradation systems fit well with the Type-2 and Type-3 pseudo-second order (PSO) kinetic models with R^2^ > 0.999. It suggested that the proposed statistical and kinetic-based modeling approach is promising support for predicting the chemical oxidation performance of organic contaminants in groundwater under the influence of multiple factors.

## 1 Introduction

The widespread use of organics in industrial production and the lack of management of organic wastewater leakage and discharge have led to an increasing threat of organic contaminants to the public safety of subsurface ecosystems (Padhi and Gokhale, 2017). Benzene, as an important aromatic compound existing in pesticide intermediates and petroleum products, is widely used as an organic solvent in the industry (Liu et al., 2010; Zhao et al., 2020). Benzene has the potential to readily infiltrate the subsurface milieu during various stages of its lifecycle, encompassing production, storage, and transportation. Its inclusion in the U.S. EPA’s National Priorities List is attributed to its well-documented carcinogenic, teratogenic, and mutagenic properties, which are observed in both its liquid and gaseous states (Xu Q. et al., 2021; Agency, 2023). Subsurface environmental exposure to benzene has become a global environmental problem (Singh and Fulekar, 2010; Padhi and Gokhale, 2014). Therefore, the rapid and precise remediation of benzene-contaminated groundwater to reduce public risk has become a widespread concern.

Among the many remediation strategies for organic compound contaminated groundwater, *in situ* chemical oxidation (ISCO) has attracted much attention for its advantages of economy and high efficiency (Matzek and Carter, 2016). At present, the sulfate radical-based advanced oxidation processes ( ${\text{SO}}_{4}^{\cdot -}-\text{AOPs}$ ) have been widely used in the remediation of organic contamination in groundwater. Persulfate (PS) is considered to be a promising oxidant, which degrades organic contaminants by producing sulfate free radicals. PS is often used in conjunction with activators to form highly active species during ISCO (Liang et al., 2008). Nano-zero-valent iron (nZVI) is an activator with strong reactivity, high catalytic activity, and reducing ability (Lominchar et al., 2018; Cabrera-Reina et al., 2020). The successful applications of nZVI in the remediation of organic compound contaminated groundwater have been reported. Zhu et al. (Zhu et al., 2016) found that PS/nZVI system effectively alleviated the ${\text{SO}}_{4}^{\cdot -}$ quenched by ${\text{Fe}}^{2+}$ in the traditional ISCO process. Song et al. (Song et al., 2019) indicated that PS/nZVI oxidized up to 80% of benzene-based contamination in a pilot scale field study. Nevertheless, the prevailing researches on PS/nZVI oxidation technology revolved in the refinement and optimization of individual parameters such as oxidant dose, activator dose, pH and temperature (Srivastava et al., 2021b). Owing to the heterogeneity and the complexity of subsurface surroundings, the outcomes attained were not universally generalizable. Consequently, it is necessary to develop a predictive approach that holistically incorporates the impacts of multiple process parameters and environmental factors on the oxidation effectiveness in a simpler and more flexible way to support the determination of the optimal process parameter combination for a specific scenario.

The traditional one-factor-at-a-time (OFAT) approach for process parameter optimization necessitates an excessive amount of time and runs to ensure precision in effect estimation, and its ability to determine the interaction between input variables and the optimal levels of various factors is limited (Sachaniya et al., 2020). The process-centered, statistics-based response surface methodology (RSM) overcomes these disadvantages. RSM is a statistical modeling method that uses multiple quadratic regression equations to fit the global functional relationship between the factors and the response values through reasonable experimental design (Amiri et al., 2019; Bahrami et al., 2019). By analyzing the response surface contour plot, the interactions among the process parameters and contaminant degradation rate in the PS/nZVI oxidation system of organic contamination in groundwater would be determined, and the optimal factor value could be predicted by regression equation (Kasiri et al., 2008). The commonly used RSM design methods include Box-Benkhen design (BBD) and Center Composite Design (CCD). It is generally believed that CCD was suitable for situations with multi-factor and continuous variables, and when the number of variables is small (three to seven variables), and BBD could reveal the interaction of multiple factors affecting the remediation effect by using fewer experimental groups than CCD (Ray et al., 2009).

However, the quadratic regression modeling, frequently employed in RSM, may prove inadequate for capturing the intricate relationships between these factors and the responses. Integrating Artificial neural network (ANN) as a modeling tool alongside RSM may further enrich our understanding of the intricate connections between inputs (characteristic factors) and outputs (degradation rate). ANN has garnered extensive utilization in diverse scientific and engineering domains for simulation and prediction purposes (Aycan DÜMencİ et al., 2021). Its ability to adapt, learn, identify, verify, and reproduce associations enables ANN to effectively interpret the interaction of highly complex factors in the remediation process of contaminated groundwater, and to simulate and predict degradation data through the analysis of characteristic parameters (Zafar et al., 2012). Currently, ANN has demonstrated its efficacy in tackling numerous challenging issues within the realm of environmental remediation, such as the prediction of the elimination of hazardous compounds from industrial wastewater and the precise management of diverse degradation processes associated with organic contaminants (Lenzi et al., 2016; Srivastava et al., 2021a).

Chemical degradation kinetic models play a vital role in providing insight into transient degradation rates and comprehending the chemical oxidation process, thereby furnishing crucial information for simulating site-specific contaminant degradation on a larger scale (Lominchar et al., 2018). At present, the pseudo-first-order kinetic model (PFO), pseudo-second-order kinetic model (PSO), and intraparticle diffusion kinetic model (IPD, W-M equation) have been demonstrated as the simplified mathematical models for simulating advanced oxidation process (Zulfiqar et al., 2019). Therefore, combining the chemical degradation kinetic model with the RSM-ANN approach may provide a deeper insight into the transient degradation processes within the system, thus furnishing a more realistic framework to guide practical applications. Currently, the RSM-ANN-kinetic approach has been successfully applied in the areas such as optimizing wastewater treatment processes (Nayak and Pal, 2020; Igwegbe et al., 2023) and evaluating catalyst performance (Fattahi et al., 2014; Kassahun et al., 2021).

Many studies have been carried out in modeling and optimizing the degradation of organic contaminants through activated PS oxidation techniques using kinetic models based on OFAT (Cabrera-Reina et al., 2020; Heidari et al., 2022; Conte et al., 2023). Other studies focused on modeling and optimizing the pollutant degradation process via activated PS using the combined approach of RSM and ANN (Zhang et al., 2018; Asgari et al., 2020; Qiu et al., 2021). However, few studies have systematically explored the effectiveness of the coupled RSM-ANN-kinetic approach in predicting the oxidation of organic contaminants in groundwater by activated PS.

In this study, the modeling approach coupling two optimization systems namely, RSM-BDD and ANN with kinetic model was innovatively implemented to optimize the benzene degradation process parameters and predict the effect of PS/nZVI oxidation system with benzene. The RSM models of four independent parameters, including PS dosage, nZVI dosage, pH, and temperature were established based on batch experiments. The simulation accuracy of the RSM models was optimized using ANN to enhance the prediction accuracy of the advanced oxidation degradation models based on statistical data. In addition, the kinetic models suitable for predicting the degradation process of benzene-contaminated groundwater by the PS/nZVI oxidation system were developed.

## 2 Materials and methods

### 2.1 Field sample

The soil and groundwater samples used in this study were collected from an abandoned pesticide factory in Jiangsu Province, China. Benzene, toluene, ethylbenzene, and xylene (BTEX) was found to contaminate the core area of the site. The soils used in this work were taken from the non-polluted area at the edge of the site, and the uncontaminated groundwater was obtained from the upstream of the contaminated site. The soil samples were dried (for a week) in a greenhouse before being screened, and collected with a particle size of less than 2 mm for later use.

### 2.2 Microcosm experiment setup

Benzene was added to the collected uncontaminated groundwater to a final concentration of 0.25 mM (equivalent to 20 mg/L). The groundwater was then aliquoted to the standard 40 mL threaded vials filled with the pre-prepared soil and capped with a Teflon/silicone gasket to prevent the volatilization of contaminants. PS (purity >98%, Sinopharm Chemical Reagent Co., Ltd., China) and nZVI (Sinopharm Chemical Reagent Co., Ltd., China) were added to the above vials according to experimentally designed concentrations of RSM-BBD and sodium hydroxide solution (0.1 M) and hydrochloric acid solution (0.1 M) were used for pH adjustment. All the microcosmic vials were kept for 12 h at the design temperature after uniform oscillation. Then the sample were filtered through a 0.45 μm membranes before analysis. The residual benzene concentration analysis was conducted using a gas chromatography-mass spectrometrometry system (GC-MS, 7890A/5975C, Agilent, United States) equipped with a capillary column (J&W Scientific DB-624 60 m × 0.25 mm × 1.4 μm, Agilent) after a purge and trap concentrator (Eclipse 4,552and4,660, OI Analytical, United States). Three parallel experiments were set in each group to eliminate experimental errors and determine the reproducibility of the results, and one blank control group was set without degradant to eliminate interference caused by adsorption. The removal rate of benzene was calculated by the following formula:

$$\text{Benzene removal efficiency}=\frac{C_{0}-C}{C_{0}}\times 100\%$$ (1)

$C_{0}$ and C were the concentrations of benzene in the system at the beginning and the end of the reaction.

### 2.3 Experimental designs with RSM method

Design Expert software (DES, Version 11.0) was used to analyze the influence of different environmental parameters on the degradation rate of benzene (response) in contaminated groundwater. A four-variable with three-level BBD experiments with three central points were designed to investigate the degradation trend of benzene in contaminated groundwater in the PS/nZVI oxidation system. Four independent variables were selected, including 1) oxidant (PS) dose, 2) activator (nZVI) dose, 3) pH, and 4) temperature, with three levels designed for each independent variable (coded values + 1, 0, and −1, see Table 1). The BBD-RSM method was used to design 29 sets of experiments under different process conditions (Table 2). These 4 independent factors were presented as $X_{1}$ , $X_{2}$ , $X_{3}$ , and $X_{4}$ , respectively for statistical computations. A quadratic model was established to fit the experimental results and show the relationship between all involved environmental variables. The quadratic model is as follows:

$$\begin{array}{c} Y=b_{0}+b_{1}X_{1}+b_{2}X_{2}+b_{3}X_{3}+b_{4}X_{4}+b_{12}X_{1}X_{2}+b_{13}X_{1}X_{3}+b_{14}X_{1}X_{4}+b_{23}X_{2}X_{3}+\\ b_{24}X_{2}X_{4}+b_{34}X_{3}X_{4}+b_{11}X_{1}^{2}+b_{22}X_{2}^{2}+b_{33}X_{3}^{2}+b_{44}X_{4}^{2}+\varepsilon \end{array}$$ (2)

**TABLE 1:** Variables and levels used in factorial design.

Levels	Variables	Low level (−1)	Medium level (0)	High level (+1)
1	Oxidizer dose (X_1_) (mM)	0.25	0.75	1.25
2	Activator dose (X_2_)	2	4	6
3	pH (X_3_)	5	7	9
4	Temperature (X_4_) (°C)	15	20	25

**TABLE 2:** BBD matrix for experimental variables and response at four factor levels.

Std	Factors								Response	
	Oxidizer dose (X_1_) (mM)		Activator dose (X_2_)		pH (X_3_)		Temperature (X_4_) (°C)		Benzene degradation (%)	
	Actual	Coded	Actual	Coded	Actual	Coded	Actual	Coded	Actual	Predicted
1	0.25	−1	2	−1	7	0	20	0	57.40	58.06
2	1.25	+1	2	−1	7	0	20	0	90.13	90.67
3	0.25	−1	6	+1	7	0	20	0	72.11	70.61
4	1.25	+1	6	+1	7	0	20	0	96.25	89.66
5	0.75	0	4	0	5	−1	15	−1	83.05	83.69
6	0.75	0	4	0	9	+1	15	−1	58.82	59.75
7	0.75	0	4	0	5	−1	25	+1	84.27	85.70
8	0.75	0	4	0	9	+1	25	+1	68.26	64.13
9	0.25	−1	4	0	7	0	15	−1	70.59	69.26
10	1.25	+1	4	0	7	0	15	−1	92.18	97.18
11	0.25	−1	4	0	7	0	25	+1	69.04	63.74
12	1.25	+1	4	0	7	0	25	+1	94.68	96.53
13	0.75	0	2	−1	5	−1	20	0	78.40	79.77
14	0.75	0	6	+1	5	−1	20	0	87.36	88.74
15	0.75	0	2	−1	9	+1	20	0	63.45	67.70
16	0.75	0	6	+1	9	+1	20	0	77.06	74.66
17	0.25	−1	4	0	5	−1	20	0	69.02	70.31
18	1.25	+1	4	0	5	−1	20	0	98.29	96.92
19	0.25	−1	4	0	9	+1	20	0	53.52	54.26
20	1.25	+1	4	0	9	+1	20	0	80.17	83.82
21	0.75	0	2	−1	7	0	15	−1	66.18	66.36
22	0.75	0	6	+1	7	0	15	−1	72.65	67.62
23	0.75	0	2	−1	7	0	25	+1	77.56	81.52
24	0.75	0	6	+1	7	0	25	+1	85.43	83.58
25	0.75	0	4	0	7	0	20	0	81.84	80.92
26	0.75	0	4	0	7	0	20	0	81.93	78.93
27	0.75	0	4	0	7	0	20	0	81.48	78.80
28	0.75	0	4	0	7	0	20	0	83.08	84.25
29	0.75	0	4	0	7	0	20	0	83.69	85.75

In the formula, Y is the response factor predicting the degradation efficiency of benzene, $b_{0}$ is the fixed response value at the design centre point, $b_{i}$ (i = 1, 2, 3, 4) represents the linear coefficient, $b_{\text{ii}}$ (ii = 1, 2, 3, 4) is the quadratic coefficient, $b_{\text{ij}}$ (i = 1, 2, 3, 4, j = 2, 3, 4) represents the effective coefficient of interactive regression, $X_{i}$ , $X_{j}$ represent independent variables, $X_{\text{ij}}$ represents the effect of interaction between independent variables, $X_{i}^{2}$ and $X_{j}^{2}$ represent the secondary effects of each independent variable, ε is the statistical error. Analysis of variance (ANOVA) was used to analyse the established model. The significance of each coefficient in the equation was determined by the F test and *p*-value, and the prediction ability of the model was evaluated through graphical analysis and numerical analysis.

### 2.4 ANN design

**FIGURE 1:**
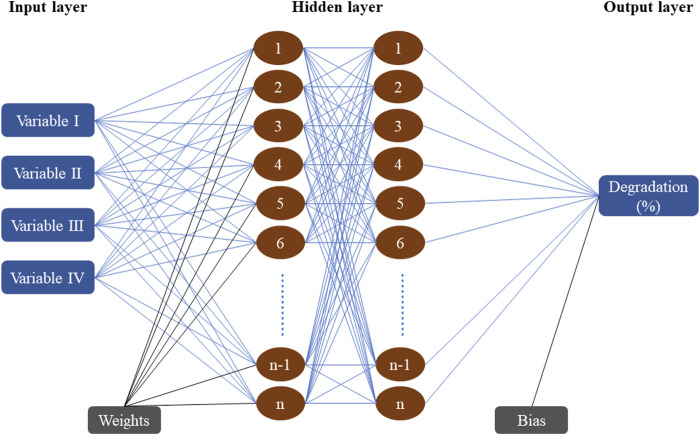
Schematic representation of neural network architecture with dual hidden layers.

The learning network used in this research was a hierarchical feedforward neural network with a back-propagation method, also known as an improved BP learning algorithm. Levenberg-Marquardt (LM) algorithm was selected for supervised learning functions to train the network because of its advantages of fast convergence and high computational accuracy (Srivastava et al., 2021a). The four variables determined by the response surface method were used as input layer neurons (04), and output layer neuron (01) was set as the response of benzene degradation rate. This research adopted the double-layer structure network with better generalization ability than the single-hidden layer structure which optimized the network structure of the hidden layer (Figure 1). Estimation of the number of hidden layers and the number of neurons in each layer was the main difficulties in optimizing the structure of the neural network, this problem was solved by applying the thumb rule (Gazzaz et al., 2012). In all sample datasets, 70% were used for training the network learning, 15% were used for forming the verification set, and the other 15% were used for forming the network test set (Taqvi et al., 2017), to cross-validate the ANN model for benzene degradation by the PS/nZVI oxidation system. The specific model parameters in the ANN were shown in Table 3. The scenario settings used to optimize the hidden layer structure of the ANN model were shown in Table 4. The development of the ANN model was completed by MATLAB 9.8.0.1323502 (R2020A).

**TABLE 3:** ANN model parameters.

Parameters	Value
Input layer neuron	04 (Oxidizer, Activator, pH, Temperature)
Output layer neuron	01 (Degradation rate)
Hidden layers	2
Transfer Function	TANSIG
Number of learning cycles	500 epochs
Performance function	MSE
Data division	70%–15% - 15% (Training -Test -Validation)
Learning function	Levenberg-Marquardt (LM)

**TABLE 4:** Performance of ANN models with different structure of hidden layer (Bold values represent the optimal hidden layer structure).

Model id	Structure of hidden layer	$R^{2}$	MSE	RMSE	MAD	MAPE
BP1	1,1	0.8887	80.1415	8.9522	12.4607	0.3697
BP2	1,4	0.9221	76.6661	8.7559	13.464	0.3802
BP3	4,4	0.914	29.655	5.4456	13.3865	0.2768
BP4	4,8	0.9202	9.655	3.1072	8.6489	0.1712
**BP5**	**8,8**	**0.9801**	**0.8243**	**0.9079**	**3.0614**	**0.1564**
BP6	8,16	0.9172	4.0153	2.003	6.958	0.1748
BP7	16,16	0.9093	18.8257	4.3388	4.081	0.2582
BP8	16,32	0.8542	60.3357	7.7676	5.8504	0.1701
BP9	32,32	0.8341	92.1841	9.6012	13.7703	0.2858

The prediction performance of the models was evaluated via error functions that include the coefficient of determination ( $R^{2}$ ), root mean square error (RMSE), mean absolute deviation (MAD), and mean absolute percentage error (MAPE), expressed as:

$$R^{2}=1-\frac{\sum_{I=1}^{N}{\left(A_{t}-F_{t}\right)}^{2}}{\sum_{I=1}^{N}{\left(A_{m}-F_{t}\right)}^{2}}$$ (3)

$$\text{MAD}=\frac{\sum_{i=1}^{n}\left|A_{t}-F_{t}\right|}{n}$$ (4)

$$\text{RMSE}=\sqrt{\frac{\sum_{i=1}^{n}{\left(A_{t}-F_{t}\right)}^{2}}{n}}$$ (5)

$$\text{MAPE}=\frac{\sum_{i=1}^{n}\left|\frac{A_{t}-F_{t}}{A_{t}}\right|}{n}\times 100$$ (6)

Where $A_{t}$ is the predicted value obtained by ANN or RSM, $F_{t}$ is the experimental/observed value, $A_{m}$ is the average of the predicted value, and *n* is the number of samples.

### 2.5 Kinetic model study

Under the optimum conditions given by RSM and ANN, the degradation kinetics of benzene with different concentrations (0.0625–1.25 mM) by the PS/nZVI oxidation system was studied. Degradation experiments were carried out on benzene solutions of specified concentrations based on optimized process parameters, and samples were collected at 20-min intervals to measure the residual benzene concentration in the system. The degradation results of benzene in the optimized experiment were modeled using the Pseudo-first order (PFO) and Pseudo-second order (PSO) kinetic models, which were widely used in the analysis of the degradation mechanism (Chowdhury et al., 2016). The diffusion mechanism of the degradation system was studied by the intraparticle diffusion (IPD) kinetic model (Zulfiqar et al., 2019).

i. PFO kinetic model

PFO kinetic model is an extensively adopted model to simulate the degradation process, which could be represented as follows:

$$\log\left(q_{e}-q\right)=\log\left(q_{e}\right)-K_{1}t/2.303$$ (7)

In the formula, $K_{1}$ (L/min) represents the degradation rate constant, $q_{e}$ (mg/g) represents the degradation amount of benzene when the system reaches equilibrium, q (mg/g) represents the degradation amount of benzene at any time point in the reaction process, and t represents the time (min). The Curve Fitting Tool of MATLAB 9.8.0.1323502 (R2020A) was used to obtain the parameter values.

ii. PSO kinetic model

Five different forms of PSO kinetic models were used to fit the degradation rate results of benzene, namely,: Type-1 PSO, Type-2 PSO, Type-3 PSO, Type-4 PSO and Type-5 PSO. These Lagergren equations are extensively used for the degradation of liquid-solid phase based on solid capacity, and these PSO kinetic formulas could be expressed as following equations (Zulfiqar et al., 2018):

$$\frac{t}{q}=\frac{1}{K_{2}q_{e}^{2}}+\frac{1}{q_{e}}t\text{ Type}-1\text{ PSO}$$ (8)

$$\frac{1}{q}=\left(\frac{1}{K_{2}q_{e}^{2}}\right)\frac{1}{t}+\frac{1}{q_{e}}\text{ Type}-2\text{ PSO}$$ (9)

$$\frac{1}{t}=\frac{K_{2}q_{e}^{2}}{q}-\frac{K_{2}q_{e}^{2}}{q_{e}}\;\text{ Type}-3\text{ PSO}$$ (10)

$$\frac{q}{t}=K_{2}q_{e}^{2}-\frac{K_{2}q_{e}^{2}q}{q_{e}}\text{ Type}-4\text{ PSO}$$ (11)

$$\frac{1}{q_{e}-q}=\left(\frac{1}{q_{e}}\right)+K_{2}t\text{ Type}-5\text{ PSO}$$ (12)

Where $q_{e}$ (mg/g) and q (mg/g) respectively represent the amount of benzene removed by the oxidant per unit weight under equilibrium and at any time in the reaction process. $K_{2}$ (g/mg min) represents the PSO chemical degradation rate constants. Using Type-1 PSO, the graph against t *versus* t/q was plotted. The slope and intercept were used to achieve the values of constant factors. In term of Type-2 PSO kinetic model, the slope and intercept were used to receive the values of constant factors after plotting the graph against 1/t *versus* 1/q. In the case of Type-3 PSO kinetic equation, the graph between 1/q *versus* 1/t was plotted and obtained the constant values using slope and intercept. By using a Type-4 kinetic model, the graph plotted against q/t *versus* q for obtaining the values of constant factors from slope and intercept. Similarly, by employing the Type-5 PSO kinetic equation, the graph was plotted against t *versus* 1/(q_e_-q) and calculate the values of K_2_ and q_e_ by using the slope and intercept.

iii. IPD kinetic model

Another kinetic theory considered the controlling factor of the reaction rate of the PS/nZVI oxidation system from the perspective of solid-liquid mass transfer. The degradation process of the benzene molecules in the system could be summarized into three steps: 1) the mass transfer of the pollutant through the outer boundary layer at the solid-liquid interface; 2) the diffusion of the liquid through the solid-phase particle channels; and 3) the oxidative degradation of the ${\text{SO}}_{4}^{\cdot -}$ on the surface of the activator (Ocampo-Perez et al., 2011). The mass transfer rate or particle diffusion rate, or the coupling effect of the two mechanisms affected the overall degradation rate of nZVI-activated PS. The IPD kinetic model obtained the prediction of the reaction process through the correlation between $t^{0.5}$ and $q_{t}$ , which was given by the following equation:

$$q_{t}=K_{\text{id}}t^{0.5}+C_{i}$$ (13)

Where $K_{\text{id}}$ (mg/g $\min^{0.5}$ ) and $C_{i}$ refer to the rate constant of IPD and the boundary layer thickness of solid particles in the system, respectively.

## 3 Results and discussion

### 3.1 Statistical analysis for BBD-RSM

The BBD-RSM successfully simulated and predicted the PS/nZVI oxidation of benzene in synthetic contaminated groundwater at each design level of the four factors. The benzene degradation rate was between 53.52% and 98.29% in the batch experiments (Table 2). The ANOVA of BBD-RSM for benzene degradation with the PS/nZVI technology showed that the predicted degradation rate of the quadratic model was highly consistent (*p* < 0.0001) with the actual degradation rate (Supplementary Table S1).

The value of determination coefficient R^2^ (0.948) indicated that the suggested quadratic equation was useful for predicting the benzene degradation rate in the PS/nZVI oxidation system within the range of experimental conditions. The R^2^-adj value (0.895) of the RSM model was close enough to the R^2^ value (0.948), indicating that the precision of the suggested response model was only slightly affected by the insignificant model terms. In general, R^2^ values would increase by adding an item which has insignificant effects (*p*-value>0.05) to the proposed model, but the adjusted R^2^ (R^2^-adj) representing significant effects would not increase (Salarian et al., 2016). In addition, the predicted R^2^ (0.792) also has favorable anastomose with the R^2^-adj (0.895) (a difference of less than 0.2 was considered acceptable), further indicating that the constructed BBD-RSM model had acceptable reproducibility.

According to the screening principle of the significance of RSM model variables (*p*-value <0.05 and F value >5), independent variables $X_{1}$ , $X_{2}$ , $X_{3}$ , $X_{4}$ , and $X_{3}^{2}$ were believed to have the most significant contributions to the accuracy of the suggested model and played the most important role in the simulation results. $X_{1}$ had the largest F value (146.20), indicating that its influence on the model was dominant compared with other factors. The measured results were fitted using a quadratic model, to achieve the following regression equations:

$$\begin{array}{c} Y=9.08+0.758X_{1}+0.280X_{2}-0.481X_{3}+0.174X_{4}-0.150X_{1}X_{2}+0.008X_{1}X_{3}+0.056X_{1}X_{4}\\ +0.080X_{2}X_{3}-0.012X_{2}X_{4}+0.132X_{3}X_{4}-0.041X_{1}^{2}-0.152X_{2}^{2}-0.322X_{3}^{2}-0.158X_{4}^{2} \end{array}$$ (14)

The prediction accuracy of the proposed model for the degradation of benzene by the PS/nZVI oxidation system was verified using four different evaluation methods. The measured value and predicted value of the degradation rate of benzene showed a high degree of consistency (Supplementary Figure S1A), which provided the most direct evidence for the great performance of the prediction model. The externally studentized residual analysis showed that the error values were normally distributed along a mathematical expectation value μ) close to zero and a constant variance ( $\sigma^{2}$ ), which confirmed the adequacy of the proposed model (Supplementary Figure S1B). In addition, the residuals of the experimental values and predicted values were uniformly distributed within a rectangular region centered at zero, indicating that the random error distribution of the proposed model was uniform and reasonable, which indirectly proved the stability of the RSM model (Supplementary Figure S1C,D).

**FIGURE 2:**
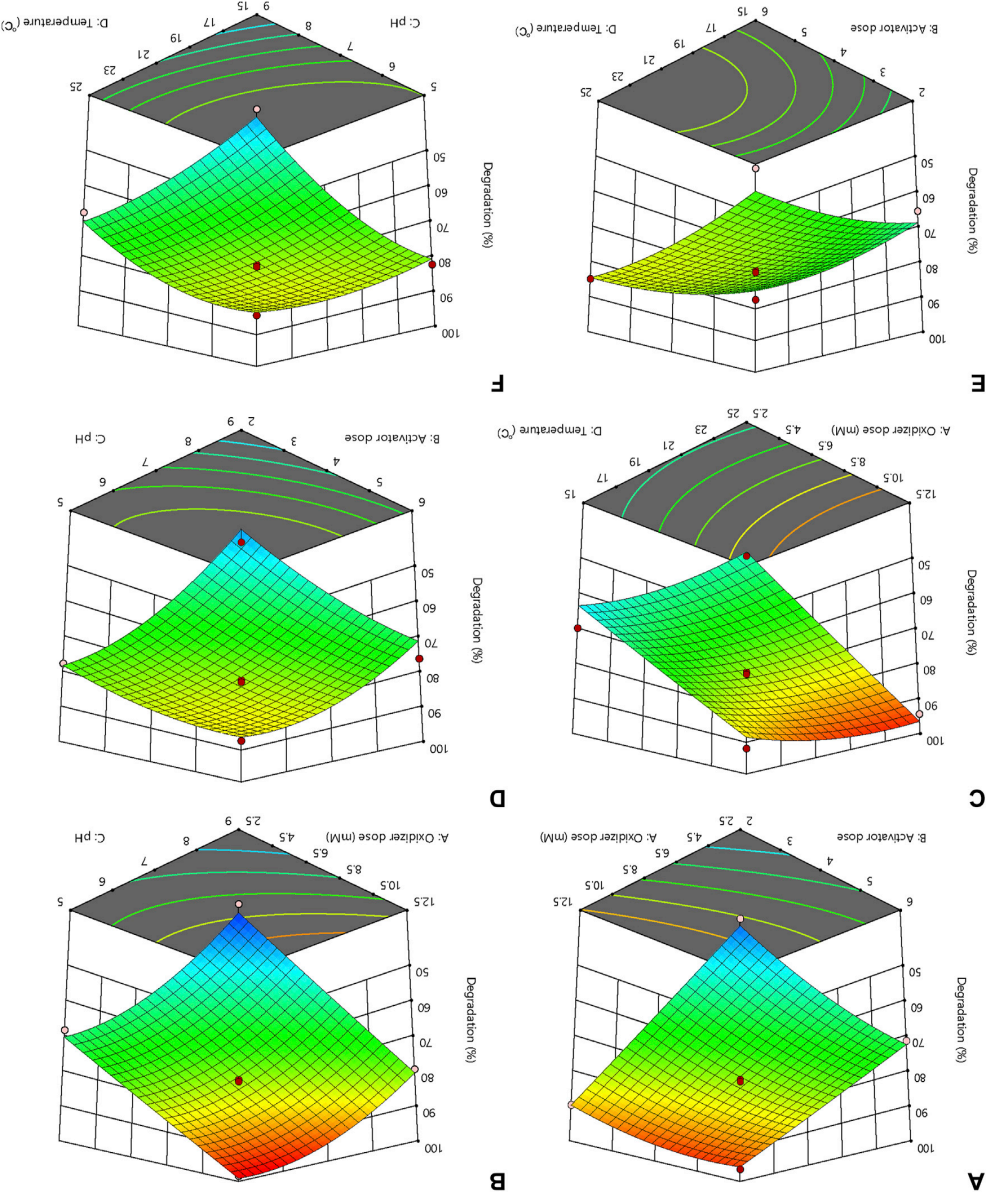
The response surface plot of benzene degradation as the function of **(A)** oxidizer and activator dosage, **(B)** pH and oxidizer dosage, **(C)** oxidizer dosage and temperature, **(D)** activator dosage and pH, **(E)** activator dosage and temperature, and **(F)** temperature and pH.

The factor interaction analysis of the response surface methodology showed that the interaction between the PS and nZVI dosages exerted the dominant effects on the benzene degradation in synthetic contaminated groundwater (Figure 2A). In the response surface of the interaction between PS and nZVI dosages, the degradation rate of benzene showed a drastic increase with the dosage of PS increasing from 0.25 mM to 1.25 mM (53.52%–98.29%), while the effect of the activator on the reaction result was more moderate. However, when the amount of activator alone was controlled to increase from a 2:1 M ratio to 6:1, a 15% increase in benzene degradation rate was also observed (Figure 2A). This may owe to the ${\text{SO}}_{4}^{\cdot -}$ played a major role in benzene degradation, and the interaction between oxidants and activators directly determined the rate of the ${\text{SO}}_{4}^{\cdot -}$ generation (Lominchar et al., 2018). Whereas, the effect of the activator on benzene degradation was much weaker, probably due to the activation potential not fully released at the set ratio with the oxidizer (Xu Z. et al., 2021). In conclusion, the PS and nZVI dosages were positively correlated with the benzene degradation rate, although PS dosage showed the more crucial role.

The response surface of the interaction between the PS dosage and pH showed that the degradation rate of benzene decreased with the transition from the acidic to the alkaline solution at a constant PS dosage (Figure 2B). Although both $H^{+}$ and ${\text{OH}}^{-}$ have an activating effect on PS, alkaline conditions inhibited the transformation of nZVI to the activating ${\text{Fe}}^{2+}$ , which suppressed the activation capacity of nZVI in the system. In addition, ${\text{OH}}^{-}$ in the system would coordinate with ${\text{Fe}}^{2+}$ and ${\text{Fe}}^{3+}$ to form compounds with no activation efficiency, and reduce the activation ability (Rodriguez et al., 2014). Therefore, under acidic conditions and high PS dosage, the PS/nZVI oxidation system was more efficient for the benzene degradation. The response surface under the interaction between the PS dosage and temperature (Figure 2C), nZVI dosage and pH (Figure 2D), nZVI dosage and temperature (Figure 2E), and temperature and pH (Figure 2F) were relatively flat or behaved as the waterfall, which indicated that the interaction of these dependent variables had no significant effect, or was mainly caused by a single factor on the benzene degradation rate in synthetic contaminated groundwater.

### 3.2 Development of ANN model

The optimization results of the topological structure of the ANN model showed that when both hidden layers contain eight neurons, the evaluation function MSE, RMSE, MAD, and MAPE have the smallest values (Table 4, Scenario BP5), which represents a perfect match between the predicted value and the actual value. However, if the number of neurons in any hidden layer exceeds 8, the error of the ANN model increased instead. It was speculated that too many neurons in the hidden layer may lead to overfitting, increase the error of the test set and further increase the overall error function (Mutasa et al., 2020). Therefore, the optimal topology structure of the ANN model for the PS/nZVI oxidation of benzene was [4-8-8-1], in which case the lowest performance evaluation functions value and the highest $R^{2}$ value (0.9801) were obtained.

**FIGURE 3:**
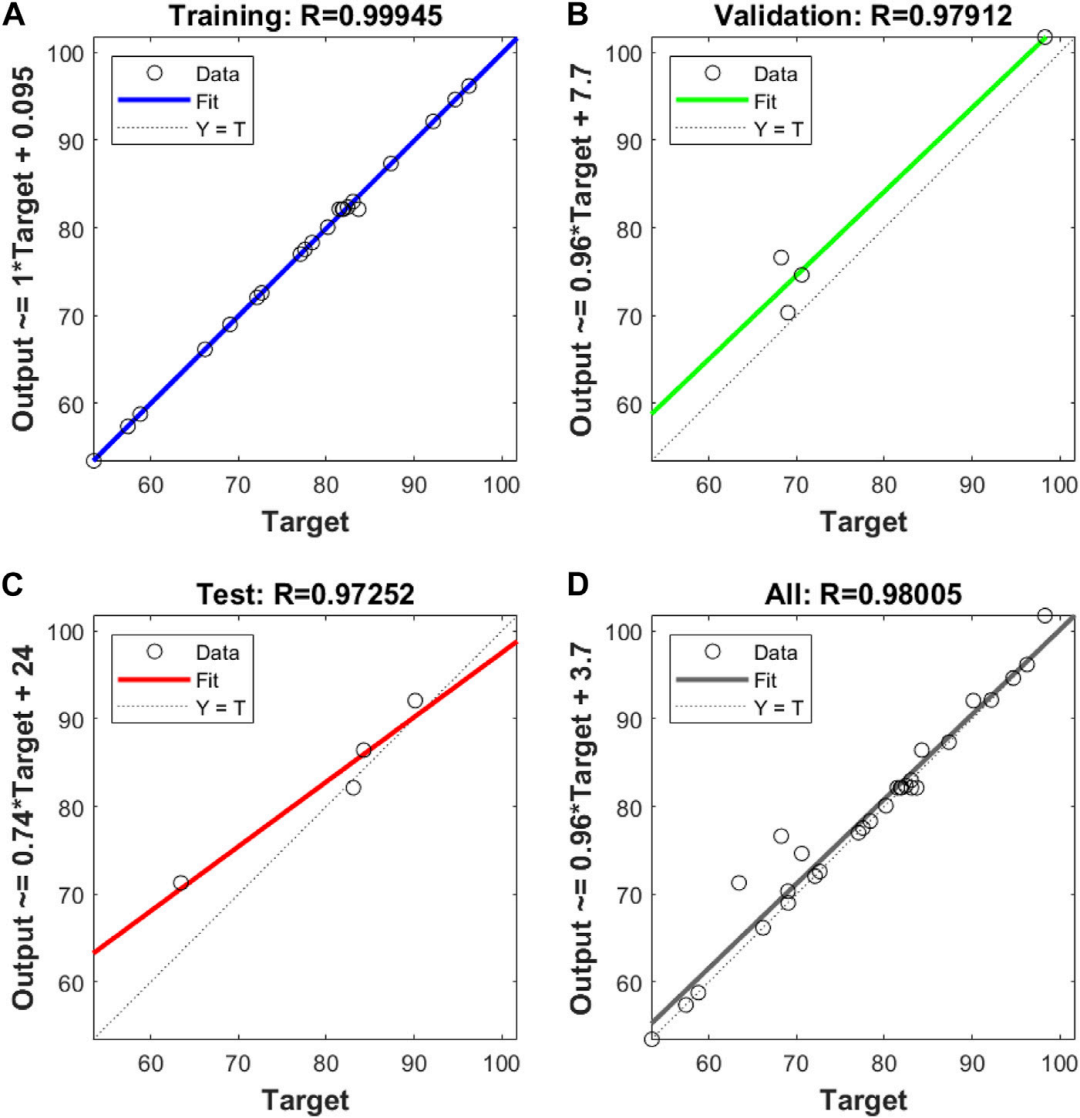
$R^{2}$ values of **(A)** training, **(B)** validation **(C)** testing, and **(D)** all data sets of ANN for benzene degradation (%) by PS/nZVI process.

The simulation of benzene degradation rates using the ANN model structured in scenario BP5 showed high goodness of fit for training, validation, and test subset (Figures 3A–C), with the $R^{2}$ values of 0.9995, 0.9791, and 0.9725, respectively. In the overall model, the ANN model fits well with the “perfect fit line” (Figure 3D), and the $R^{2}$ value (0.9801) was slightly improved compared with that of the RSM model (0.948). Therefore, the developed cascade forward ANN model can be successfully used to simulate and predict the PS/nZVI oxidation of benzene in contaminated groundwater.

### 3.3 Comprehensive evaluation comparison between RSM and ANN models

**FIGURE 4:**
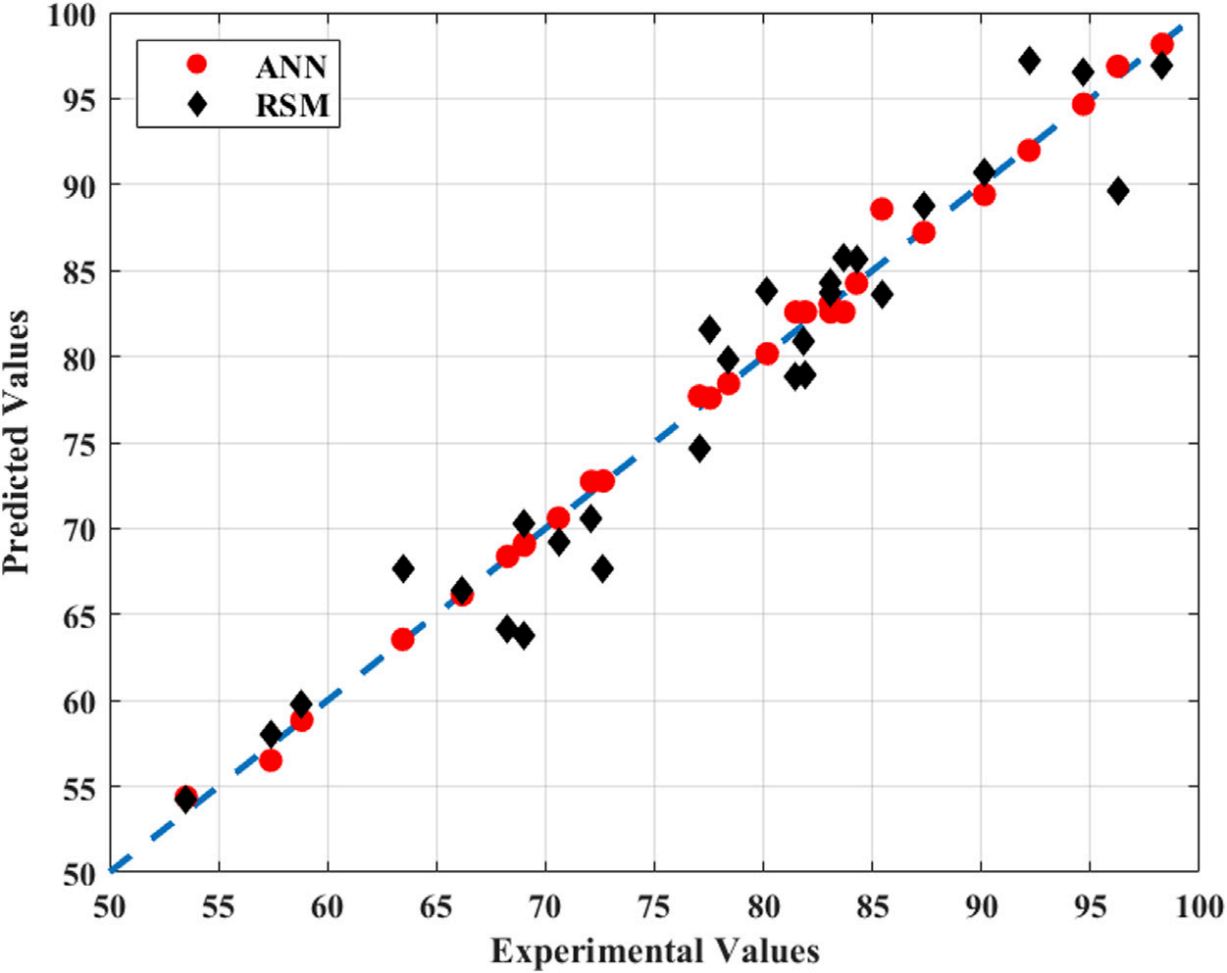
Comparison of experimental results with ANN/RSM predicted results.

The empirical modeling tool ANN displayed better prediction ability than RSM in the simulation and prediction of the PS/nZVI oxidation process of benzene in contaminated groundwater (Figure 4). We compared the observed values with the predicted values of the quadratic model obtained by RSM and the trained ANN model, respectively. Their predicted performance parameters were analyzed and the deviation of the calculated values from the actual values of the two models was plotted. Compared with the RSM, the predicted value distribution of ANN was closer to the actual value. The evaluation function values for ANN were lower, and the values of the $R^{2}$ were closer to 1, which were signs of a well-fitted model (Supplementary Table S2). The highly predictive performance of the ANN was due to its ability to extract the basic interaction between dependent variables and independent variables with high accuracy without considering the degree of nonlinearity between variables, whereas the RSM only allowed fitting data based on mathematical equations (Lopez et al., 2017). It is generally accepted that RSM is usually used for the whole process of industrial system design starting from the experimental design. Its advantage reflects in putting forward more credible suggestions on the optimization of process parameters (Jiang et al., 2020). By contrast, ANN may be more suitable for processing massive experimental data, and focus on providing and establishing more detailed interrelationships between independent variables and dependent variables (Kasiri et al., 2008). Therefore, ANN may be a more powerful and flexible empirical modelling tool for remediation simulations of groundwater contaminated by organic compounds.

### 3.4 External validation of the model

Three groups of process parameters recommended by the proposed models that should completely degrade benzene in synthetic contaminated groundwater were selected and the predictive ability of the proposed models was externally validated by replicated experiments (Supplementary Table S3). The results showed that under the condition of complete degradation of benzene predicted by the RSM model, the measured degradation rates of the three experimental groups were 100%, 99.87%, and 98.79%, respectively, and the predicted benzene degradation rates by the trained ANN (BP5, which with the best predictive performance) were 99.86%, 99.98%, and 99.10%, respectively. The agreement between the experimental and predicted values indicated that it was feasible to use the proposed model to simulate the actual degradation situation. The MSE between the predicted and measured values of the two models were 0.4937 (RSM) and 0.0426 (ANN), respectively, which indicated that the simulation errors of both models were acceptable in the external validation phase, but ANN still outperformed RSM.

### 3.5 Reaction kinetic studies

**FIGURE 5:**
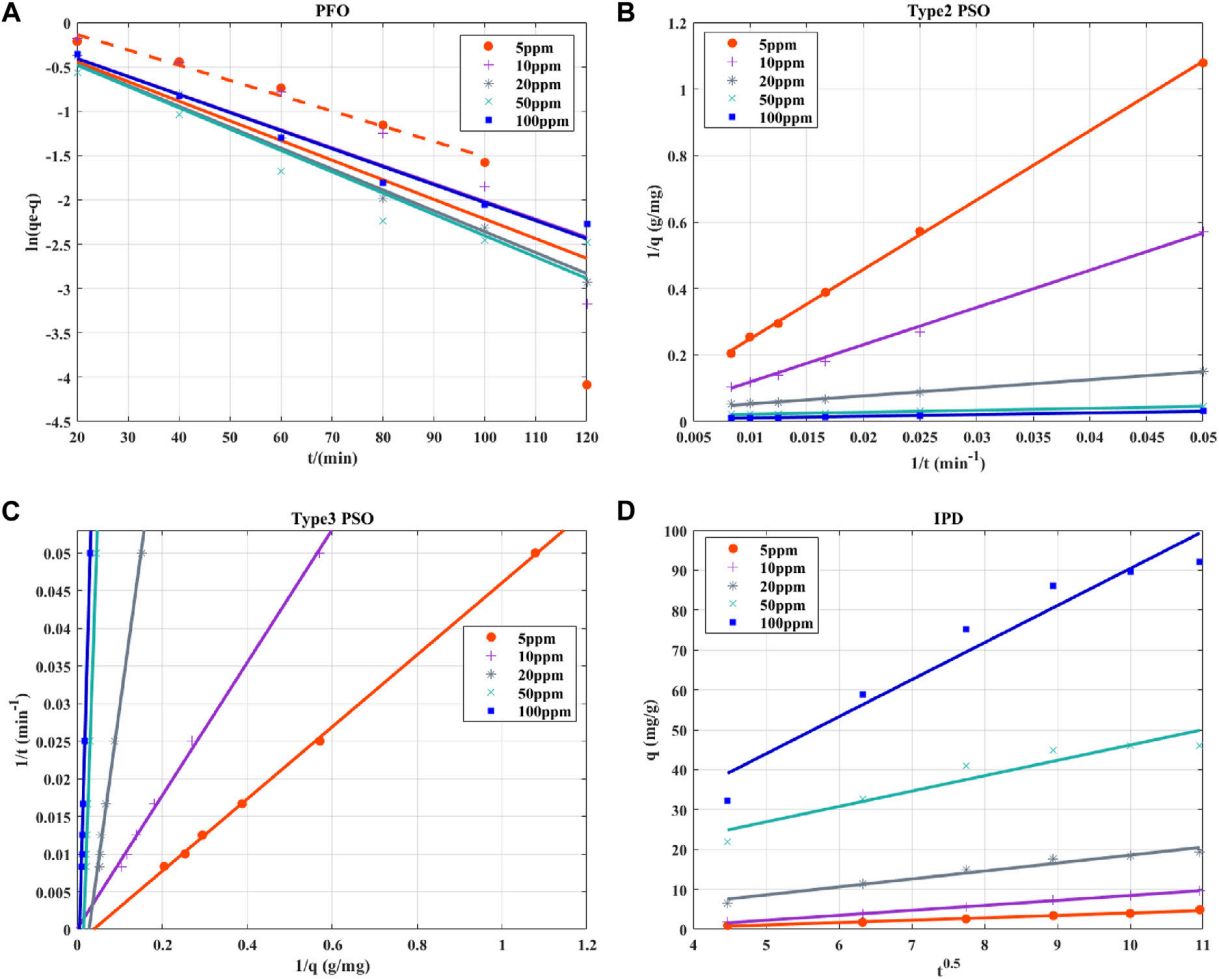
**(A)** PFO, **(B)** Type-2 PSO, **(C)** Type-3 PSO and **(4)** IPD kinetic models for benzene removal at optimized conditions given by suggested model.

The kinetic parameters ( $q_{e}$ , $K_{1}$ and $K_{2}$ ) and correlation coefficients ( $R^{2}$ ) of benzene degradation in synthetic contaminated groundwater by the PS/nZVI oxidation system were determined by linear regression method (Table 5). The $R^{2}$ values of different types of kinetic models were distributed between 0.3982 and 0.9995, and all types of kinetic models fit the experimental data well except for very few scenarios (e.g., Type-1 and Type-4 PSO at 10 mg/L benzene). The linear fitting diagram of the kinetic model showed that the fitting curve of the PFO deviated significantly from the experimental results (Figure 5A), and the parameter values of the PFO model also showed no significant regularity during the increase of benzene concentration from 0.0625 mM to 1.25 mM (Table 5), indicating that the PFO kinetic model may not be a reasonable model to explain the benzene degradation process. Type-2 and Type-3 PSO kinetic models showed the best fitting with the observed data of benzene degradation within the focused benzene concentration range of 0.0625–1.25 mM (Figures 5B,C), with $R^{2}$ values exceeding 0.99. The relationship between benzene degradation concentration *versus* time was more appropriately explained using these two types of PSO kinetic models, and they were fitted using 1/t *versus* 1/q or 1/q *versus* 1/t as the independent and dependent variables, respectively. Meanwhile, the instantaneous reaction rate models for different benzene initial concentrations can be selected from the Type-2 and Type-3 PSO kinetic models in Table 5. In addition, the results showed that the equilibrium benzene degradation amount ( $q_{e}$ ) in PSO kinetic models were positively correlated with benzene concentration, and the rate constant K showed a trend of gradual increase with the pollutant concentration. The trend of the K value was opposite to the phenomenon observed by Zulfiqar et al. (Zulfiqar et al., 2019). This may be attributed to the increase in benzene concentration, which leads to higher collision probability between free radical particles and benzene molecules in the system, resulting in improved reaction rate (Shuchi et al., 2021).

**TABLE 5:** PFO, Type-1 PSO, Type-2 PSO, Type-3 PSO, Type-4 PSO, Type-5 PSO, and IPD kinetic parameters for benzene in groundwater degradation onto PS/nZVI process.

Kinetic models	Parameters	Benzene concentrations				
		5 mg/L	10 mg/L	20 mg/L	50 mg/L	100 mg/L
PFO	$K_{1}\left(\min^{-1}\right)$	0.07630	0.06455	0.05910	0.04733	0.4525
	$q_{e}\;\left(\text{mg}/g\right)$	2.665	1.975	1.195	1.355	0.9437
	$R^{2}$	0.7632	0.9084	0.995	0.9286	0.9759
Type-1 PSO	$K_{2}\left(\min^{-1}\right)$	5.08E-05	1.27E-05	4.56E-04	5.13E-04	1.13E-04
	$q_{e}\left(\text{mg}/g\right)$	3.4321	6.2069	21.3873	40.3136	74.6969
	$R^{2}$	0.8385	0.5034	0.9827	0.9895	0.9699
Type-2 PSO	$K_{2}\left(\min^{-1}\right)$	7.43E-05	3.95E-06	3.31E-04	4.00E-04	6.53E-04
	$q_{e}\left(\text{mg}/g\right)$	25.3872	150.3307	35.1989	64.4745	175.5618
	$R^{2}$	0.9995	0.9978	0.9964	0.9927	0.9886
Type-3 PSO	$K_{2}\left(\min^{-1}\right)$	7.35E-05	4.69E-08	3.26E-04	3.93E-04	6.19E-04
	$q_{e.}\left(\text{mg}/g\right)$	25.5277	137.0321	35.4286	64.8468	179.1892
	$R^{2}$	0.9995	0.9978	0.9964	0.9927	0.9886
Type-4 PSO	$K_{2}\left(\min^{-1}\right)$	4.94E-05	9.92E-06	3.76E-04	4.05E-04	8.12E-04
	$q_{e}\left(\text{mg}/g\right)$	30.8333	97.2306	33.5975	64.272	161.9772
	$R^{2}$	0.821	0.398	0.9441	0.9489	0.8721
Type-5 PSO	$K_{2}\left(\min^{-1}\right)$	2.33E-05	5.55E-05	0.000267	0.000478	0.000124
	$q_{e}\;\left(\text{mg}/g\right)$	44.03	43.48	52.69	61.56	141.2
	$R^{2}$	0.9965	0.9975	0.9436	0.9777	0.9796
IPD	$K_{\text{id}}\left(\text{mg}/g\;\min^{0.5}\right)$	0.6002	1.242	1.995	3.851	9.334
	$C_{i}$	−1.937	−3.97	−1.389	7.607	−3.024
	$R^{2}$	0.9866	0.9979	0.9632	0.9085	0.9373

In the IPD kinetic model, as the initial benzene concentration increased from 0.0625 mM, the value of diffusivity constant $K_{\text{id}}$ increased from 0.6002 to 9.334 mg/g $\min^{0.5}$ (Table 5). This may be due to the stronger reaction driving force induced by the higher benzene concentration and thus affected the mass transfer rate in the reaction process (Cheung et al., 2007). However, it has been reported that the linear curves of negative boundary layer thickness ( $C_{i}$ ) and the linear curve of $q_{t}$ *versus* $t^{0.5}$ would not exceed zero at all concentrations (Abdelwahab and Amin, 2013). The IPD model was considered to be unable to reasonably explain the mechanism of the PS/nZVI oxidation technology due to abnormality in the fitted $C_{i}$ values (Figure 5D). Therefore, type II and type III PSO maybe the reasonable models to explain the benzene degradation process by the PS/nZVI oxidation system.

## 4 Conclusion

In this work, the modeling approach coupling BBD-RSM and modified ANN with kinetic model was implemented to optimize the benzene degradation process parameters and predict the effect of PS/nZVI oxidation system. The results indicated that the modeled optimum levels of variables were 1.45 mM PS with a Na^+^/nZVI molar ratio of 4:1 at pH 3.9°C and 21.9°C, under which 0.25 mM of benzene would theoretically be completely removed. The ANN model had better prediction performance compared with RSM, due to its strong nonlinear fitting ability. The structure of the hidden layer and the number of neurons contained in each layer significantly affected the predictive performance of the ANN. By employing the thumb rule during the test, we found that when the structure of hidden layers was [8-8], the evaluation indices of the ANN reached the optimal level. Furthermore, the developed degradation reaction kinetics showed that Type-2 and Type-3 PSO kinetic models were more suitable to explain the benzene degradation process by the PS/nZVI oxidation system. Our study is expected to provide a new approach for modeling and optimization of chemical oxidative remediation of organic contamination in groundwater.

## Data Availability

The original contributions presented in the study are included in the article/Supplementary Material, further inquiries can be directed to the corresponding authors.
