# Supplementary material for: Modeling and optimization study on degradation of organic contaminants using nZVI activated persulfate based on response surface methodology and artificial neural network: a case study of benzene as the model pollutant
Source: Front Chem. 2023 Oct 19;11:1270730. doi: 10.3389/fchem.2023.1270730 (PMC10620510; doi:10.3389/fchem.2023.1270730)

Modeling and optimization of degradation of organic contaminants using nZVI activated persulfate based on response surface methodology and artificial neural network: A case study of benzene as the model pollutant

Moye Luo [1,2], Xiaodong Zhang [2], Tao Long [2], Sheng Chen [3], Manjun Zhan[4], Xin Zhu [2]*, Ran Yu [1]*

Supplementary material

Table S1. ANOVA for response surface coefficient of quadratic model.

Source	Sum of Squares	df	Mean Square	F-value	p-value	
Model	11.97	14	0.8550	18.11	< 0.0001	Significant
X_1-Oxidizer dose	6.90	1	6.90	146.20	< 0.0001	
X_2-Activator dose	0.9413	1	0.9413	19.94	0.0005	
X_3-pH	2.78	1	2.78	58.80	< 0.0001	
X_4-Temperature	0.3628	1	0.3628	7.68	0.0150	
X_1X_2	0.0895	1	0.0895	1.90	0.1902	
X_1X_3	0.0002	1	0.0002	0.0053	0.9432	
X_1X_4	0.0123	1	0.0123	0.2612	0.6172	
X_2X_3	0.0256	1	0.0256	0.5426	0.4735	
X_2X_4	0.0006	1	0.0006	0.0121	0.9140	
X_3X_4	0.0692	1	0.0692	1.47	0.2460	
X_1^2	0.0111	1	0.0111	0.2353	0.6351	
X_2^2	0.1488	1	0.1488	3.15	0.0976	
X_3^2	0.6732	1	0.6732	14.26	0.0020	
X_4^2	0.1618	1	0.1618	3.43	0.0854	
Residual	0.6610	14	0.0472			
Lack of Fit	0.6504	10	0.0650	24.57	0.0037	Not significant
Pure Error	0.0106	4	0.0026			
Cor Total	12.63	28				
R^2 = 0.948<br>Adjusted R^2 = 0.895<br>Predicated R^2 = 0.792<br>C.V % = 2.47<br>Adequate precision=15.86						

Table S2. Predictive capability measures of RSM and ANN models.

Performance parameters	ANN	RSM
R^2	0.9801	0.9477
RMSE	0.9079	2.7748
MAD	3.0614	3.2165
MAPE	0.1564	0.2980

Table S3. Experimental value and ANN verification under the premise of optimum value prediction of RSM.

Std	Oxidizer dose (X_1) (mM)	Activator dose (X_2)	pH (X_3)	Temperature (X_4) (°C)	Benzene degradation (%)		
					$RSM_{pre.}$	Exp.	$ANN_{pre.}$
1	1.33	3.75	4.86	24.16	100.00	100.00	99.86
2	1.45	4.00	3.90	21.91	100.00	99.87	99.98
3	1.51	3.36	4.13	16.00	100.00	98.79	99.10

Fig. S1. (a) Predicated vs. actual, (b) Normal plot of residual, (c) Residual vs. run, and (d) Residual vs. predicated for percentage of benzene degradation.

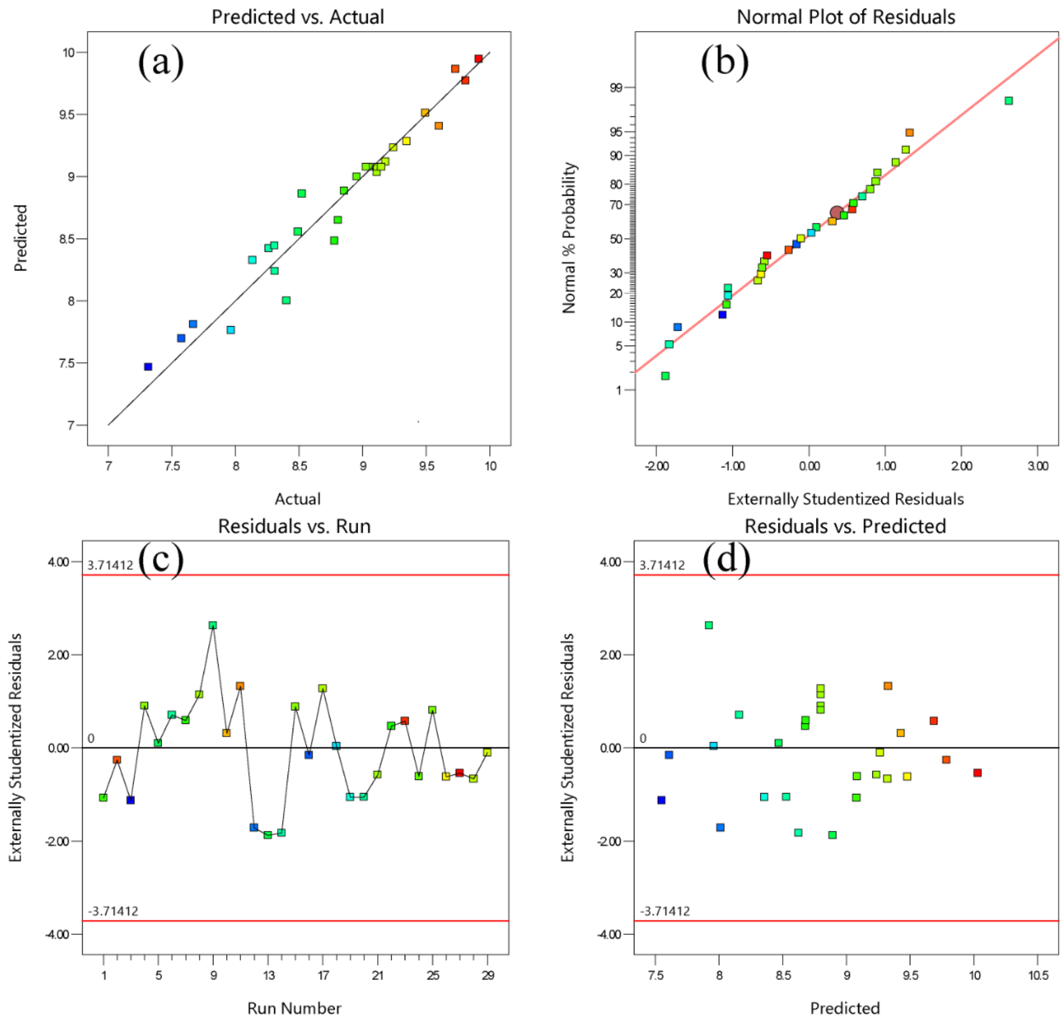

Supplement: Supplementary file 1 [file DataSheet1.pdf]
